# A Novel Method Testing the Ability to Imitate Composite Emotional Expressions Reveals an Association with Empathy

**DOI:** 10.1371/journal.pone.0061941

**Published:** 2013-04-23

**Authors:** Justin H. G. Williams, Andrew T. A. Nicolson, Katie J. Clephan, Haro de Grauw, David I. Perrett

**Affiliations:** 1 Division of Applied Health Sciences, University of Aberdeen Medical School, Clinical Research Centre, Royal Cornhill Hospital, Aberdeen, United Kingdom; 2 Perception Laboratory, School of Psychology and Neuroscience, University of St Andrews, St Andrews, Fife, United Kingdom; Ghent University, Belgium

## Abstract

Social communication relies on intentional control of emotional expression. Its variability across cultures suggests important roles for imitation in developing control over enactment of subtly different facial expressions and therefore skills in emotional communication. Both empathy and the imitation of an emotionally communicative expression may rely on a capacity to share both the experience of an emotion and the intention or motor plan associated with its expression. Therefore, we predicted that facial imitation ability would correlate with empathic traits. We built arrays of visual stimuli by systematically blending three basic emotional expressions in controlled proportions. Raters then assessed accuracy of imitation by reconstructing the same arrays using photographs of participants’ attempts at imitations of the stimuli. Accuracy was measured as the mean proximity of the participant photographs to the target stimuli in the array. Levels of performance were high, and rating was highly reliable. More empathic participants, as measured by the empathy quotient (EQ), were better facial imitators and, in particular, performed better on the more complex, blended stimuli. This preliminary study offers a simple method for the measurement of facial imitation accuracy and supports the hypothesis that empathic functioning may utilise motor control mechanisms which are also used for emotional expression.

## Introduction

Facial emotional expression was considered by Darwin [1] to be universally constant and largely innate. Consequently, models have been proposed [2], [3] that describe the vast domain of emotional expression in terms of interaction between typically six “basic” emotions. More contemporary perspectives appreciate that one of the most distinctive qualities of human social communication is that people utilise an extensive repertoire of facial actions in a variable way across cultures to communicate emotion in a flexible way according to its context [4], [5]. For facial expressions to be culturally shaped they need to be imitated [6], suggesting that imitation plays an important role in their development.

Imitation is distinguished from mimicry which has been studied using electromyography [7], and involves the triggering or release of a previously learnt motor program. Unlike imitation, mimicry does not provide a mechanism to modify and expand the existing repertoire of facial expressions. Imitation is characterised by a capacity to enact an action from seeing someone else do it [8], requiring a cognitive representation of *how* an action is performed [9]. Therefore, whilst mimicry may utilise a shared experience of emotion encoded as primary representations encoded in sensorimotor systems [8]–[11] imitation also requires a secondary representation in the form of an intention or motor plan for that same action [12]. In this respect, facial imitation may draw upon similar mechanisms to those serving empathy, which is also concerned with both the communication of emotion and a secondary representation of that emotion which enables understanding [13]. This argument is closely tied to the simulation model of empathy, which suggests that the empathiser may use his or her neural systems for imitating actions ‘off-line’ to imagine and understand the experiences of others [14], [15], and the Perception-Action model of empathy [16], which argues that empathy relies upon the perception-action coupling mechanisms that we consider necessary for imitation. These cognitive models of empathy propose reliance on the ‘mirror neuron’ system [14], [17], [18] which is also thought to be important for imitation [19].

Nevertheless, despite so much theoretical argument hypothesising a relationship between empathic traits and imitation ability [14], [15], [16], supporting empirical evidence is limited [20]. Some evidence comes from research in autism, where problems with both empathy and imitation co-occur [21], and a poor repertoire of facial expressions has diagnostic value [22], [23]. This contrasts with the art of acting which concerns itself with the effective portrayal of complex mental states through subtle control over actions. One reason for a lack of evidence may be that imitation of emotionally communicative action has been relatively little researched. Given that so much emotion is expressed through facial action, this would principally concern research into facial imitation. Much interest has been shown in neonatal imitation [24] but many argue that this is a non-specific response to social interaction [25] or a primitive reflex [26]. Studies of facial imitation in older populations utilising basic emotions [27]–[29] are unlikely tests of empathy given that recognition of basic emotions is categorical [30], and is achieved by one-year-olds [31]. If empathy is a measure of the complexity of action planning or understanding that underpins an emotional state, then a basic expression will require only a basic level of empathy. For previously learnt expressions, only recognition and then execution of a behaviour pattern is required. Tasks involving novel sequences or novel facial actions may offer stronger tests of imitative ability [29], [32], [33] but seem unlikely measures of emotional understanding.

We wished to test the hypothesis that the ability to imitate plausibly emotionally communicative facial expressions would be associated with empathic qualities. For this we required a measure of imitation accuracy at distinct, non-arbitrary levels of imitation difficulty. In the non-human primate literature, imitation is most reliably tested using two-action methods [34]. Two-action methods show each of two distinct approaches to solving the same problem to separate, matched subject pools. Participants are then observed solving the problem to see if they are more likely to utilise the method demonstrated to them than the one demonstrated to others. This approach therefore determines whether individuals show imitation according to which of two possible behaviours they saw. It therefore relies on whether discrimination between two modelled alternative behaviours, is maintained when that group of behaviours is copied. For this study, we sought to adapt this method to ask how well, rather than if, people could imitate by asking how different actions had to be from one another before imitators could successfully show evidence of discriminating between them in their copies. We assessed how closely a set of imitated actions corresponded to a set of quantitatively related modelled actions by systematically varying the actions in their degree of similarity. This would establish the threshold at which copies could no longer be discriminated from one another and so provide a measure of imitation ability. This approach to facial imitation required us to synthesise novel facial expression stimuli which were measurably different from one another along a continuum.

## Methods

### Participants

Participants were typical adults recruited by word of mouth. Written consent was obtained from volunteers before participation. The consent procedure and study as a whole was approved by the Ethics Review Board of the College of Life Sciences and Medicine of the University of Aberdeen. The experimental programme was run in an HTML/JavaScript interface. Nine men and 15 women participated after one female outlier with particularly poor imitation (error score mean >3SD) was removed from analysis (data normalised Shapiro-Wilk statistic = 0.950, df = 24, p>0.267). These participants were aged between 16 and 26 years (mean = 21.04±2.84[SD]). Men were slightly older than women but not significantly so (male mean = 22.44±2.88[SD]; female mean = 20.2±2.54[SD]; t = 2.00, df = 22, p = 0.059). Empathy quotient ranged from 13 to 64 (mean 38.1±12.8[SD]) and differences between sexes were not significant (male mean = 33.8±12.7[SD]; female mean = 40.7±12.6[SD]; t = 1.31, df = 22, p = 0.205). Distribution of EQ, age and imitation error scores were all normally distributed according to inspection of data and Shapiro-Wilk statistic (all p>0.189).

### Materials

**Figure 1:**
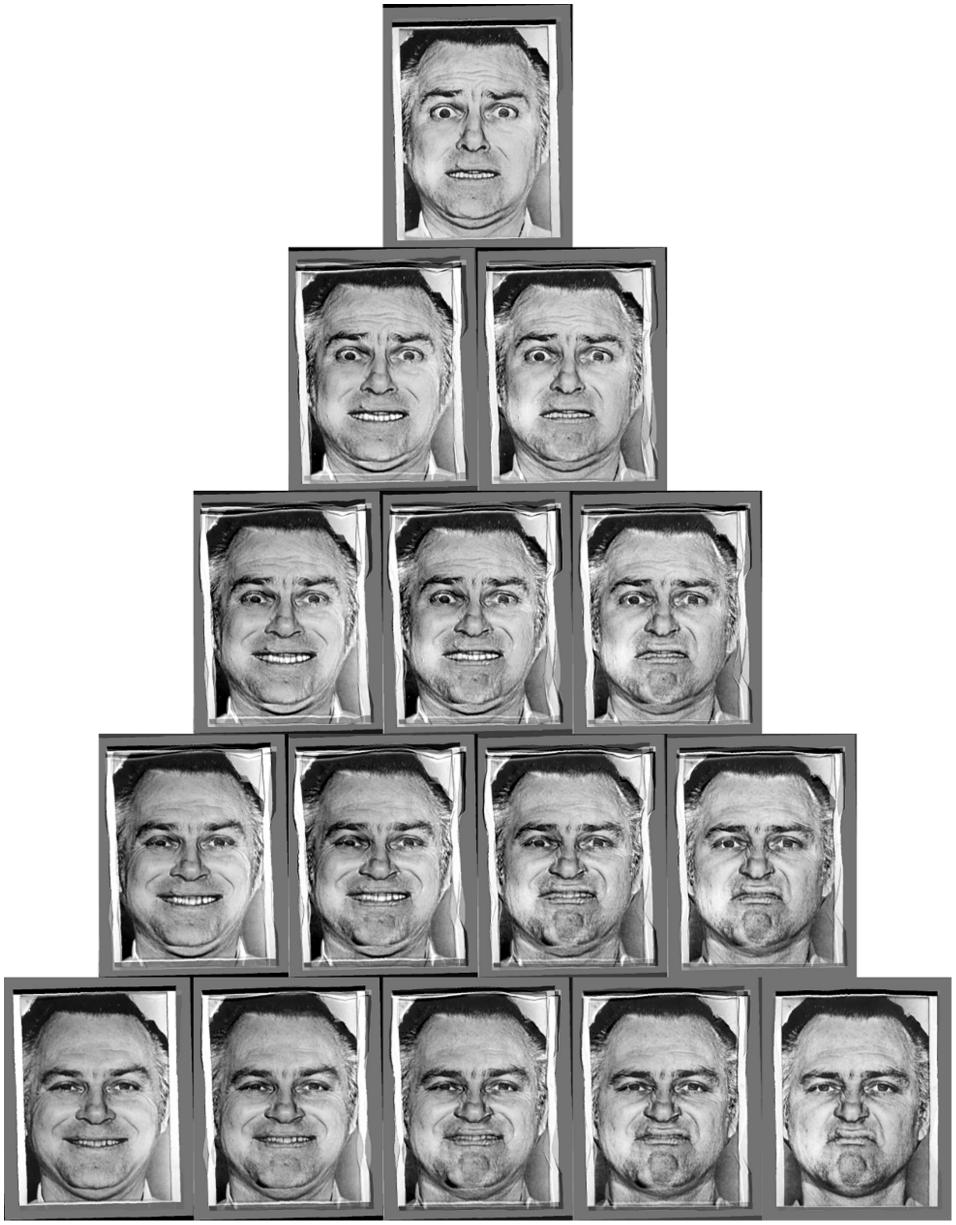
Array of emotional stimuli generated arranged in a triangle to show patterns of continuous variation. This image was created from photographs of ‘JJ’ [36], original image is ©Paul Ekman, reproduced with permission.

We created two stimulus arrays of composite-emotions, each incorporating 15 facial stimuli arranged in the form of an equilateral triangle (Figure 1). The vertices represent three (of six) basic emotions, whilst the stimuli at intermediate positions consist of blends of the basic emotions, thus making the emotional content of the expression more ambiguous. The extremes were caricatured to 110%, and the remaining stimuli were placed recursively at midpoints, exaggerated to contain varying proportions of the three basic emotions up to a constant cumulative expression level of 110%, calculated as the Euclidean distance from the neutral expression in x-y-z face space (where x-y-z are three perpendicular axes representing the three basic emotions). Thus, all stimuli in the array were arranged along a spherical surface (with radius r = 110%) in x-y-z face space, centred on the neutral expression. The allocation of emotions to the two triangles was based on the FEEST Hexagon (Facial Expression of Emotion: Stimuli and Tests), which arranges the six basic emotions on the points of a hexagon such that more confusable expressions are adjacent to one another. This served to maximise contrast between opposing emotions in each triangle. The triangles consisted of: Sadness-Anger-Surprise (SAS) and Fear-Happiness-Disgust (FHD).

**Figure 2:**
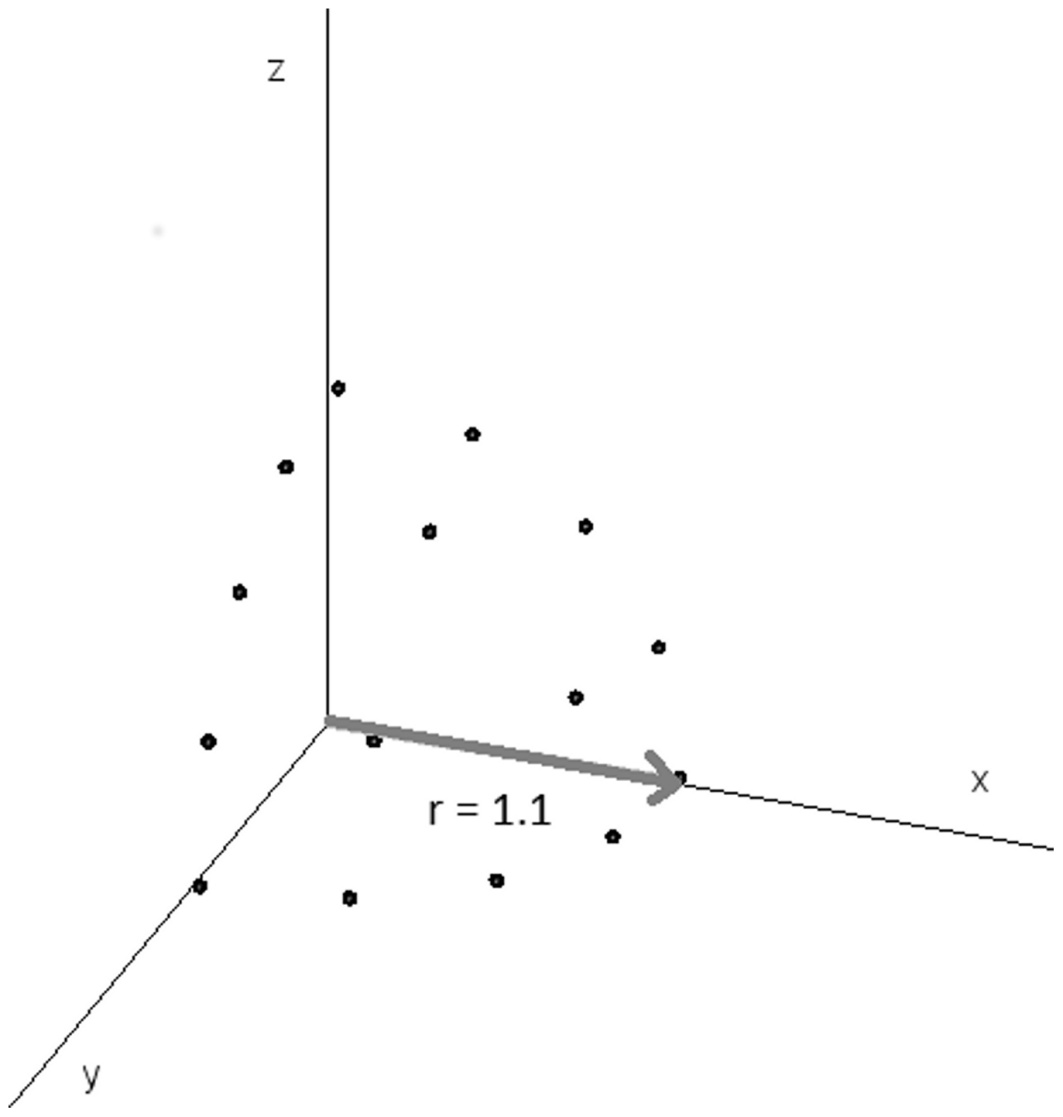
Array of points on surface of a sphere with distance from vertex determining relative proportions of emotion in each blend.

Facial stimuli were derived from the ‘JJ’ set [35] of seven greyscale photographs – one for each single emotion and one neutral expression. Image transformation techniques were used whereby image shape and lightness were warped to express varying proportions of the difference between the neutral image (N) and the emotion image (E_i_), where shape difference is computed as the shift in x-y coordinates of a set of feature landmarks [36], [37]:
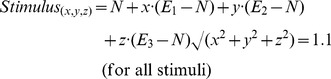



The relative percentage contribution of each shape transform to each stimulus within the array was then determined by translating its position within the array to a vector located on a three-dimensional spherical surface with each axis representing a single emotion and the x,y,z coordinates of the central point being a completely neutral expression at (0,0,0) (Figure 2). The singular emotions (i.e. purely fear, purely anger) were slightly caricatured, taken to 110% of the original expression. Using these techniques, we created two arrays of synthetic emotional stimuli, where each emotion was expressed proportionally to its inverse-distance in the array from the location where it was most expressed.

### Procedure

Participants were seated in front of a computer and asked to imitate the displayed stimuli. After a training block of five images (comprising three basic emotion vertices, the neutral expression, and one composite emotion), each stimulus from both arrays of 15 images was shown once, in three blocks of 10 images, each to constitute a ‘run’. No two adjacent images from the same array were in the same block, and hence the blocks were balanced with respect to content of vertex (basic emotion) and compound expressions. Within each block, images alternated between the two arrays; otherwise the order of the images and blocks was randomised. For each trial, a fixation cross was followed by the image for 10 seconds, after which a sound clip counted 3–2–1, and a photograph was taken; then the next image appeared. A webcam (Logitech HD C310) mounted on the top of the screen was used for photocapture. The run was then repeated. Participants were randomly allocated to receive visual feedback from the webcam, i.e., a real-time view of themselves, on either the first or second run, with the relevant part of the screen being masked in the remaining run. Therefore, each participant imitated each image four times. Half the participants received visual feedback during the first half of the experiment and half received it during the second half. In addition, participants completed the empathy quotient (EQ); which is a 60-item self-report questionnaire that can reliably differentiate participants according to empathic traits [13].

### Scoring

One researcher printed the photocaptured attempts at imitation and noted on the back of each the position in the array of the corresponding stimulus. A second researcher, blind to the correct source image, judged the position in the array for each response that they thought had served as the model. The scorer could not select a position already occupied without moving the response already occupying that position. Once the scorer was satisfied that he or she had achieved the arrangement of responses that best matched the stimulus array, the arrangement was unblinded and scored. A score of ‘0’ was allocated to every image placed in the correct position. Otherwise the score reflected the distance between the placement and the correct position, counted according to the minimum number of steps between the erroneous and the correct positions on the triangle. Therefore, the highest score that could be gained for a single image was 4, since no two points on the triangular array are more than four steps apart. Each participant was scored twice, to improve reliability. A sample of scores for 16 sets of triangular arrays were rated by both raters.

## Results

The mean total error score for an individual was 3.97 steps per array of 15 stimuli and 0.265 for each item (34.6% of trials were scored as being correct and a further 49.9% were within 1 step of being correct). This score is substantially higher than would be expected for an error rate from random performance (2.200+/−0.982).

Effects of EQ and visual feedback were investigated with a repeated measures ANOVA. EQ scores were categorised into High or Low scores according to whether they were above or below the median score (low <44). Within-participant factors were the arrays of emotions (two levels) and Block (two levels). High/Low EQ, task order (visual feedback provided during first or second block) and sex were included as a between participant factors and age was included as a covariate. This revealed a main between-subject effect of EQ (F(1, 15) = 7.79, p = 0.014, *η* ^2^ = 0.342. There were trends towards significant interactions between task order and sex (F(1, 15) = 3.87, p = 0.068, *η* ^2^ = 0.205) and task order and High/Low EQ (F(1, 15) = 3.38, p = 0.086, *η* ^2^ = 0.184) but no other main effects or interactions were significant or close to significant (all F<3, p>0.1).

**Figure 3:**
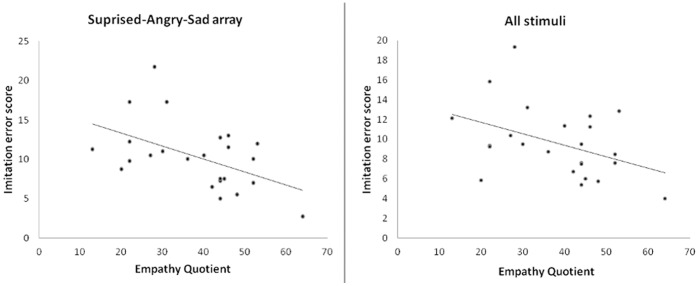
Scatter plots showing relationship between empathy quotient and imitation ability.

To explore the relationship between EQ and emotion array more closely, we examined correlations between EQ and error scores for the two emotion arrays separately and combined. EQ correlated negatively with the combined error score (Pearson r = −0.420, p = 0.041, n = 24) and separately, EQ correlated negatively with the error score for the SAS array (r = −0.500, p = 0.013, n = 24) but not with the FHD array (r = −0.253, p = 0.234, n = 24) (Figure 3). These correlations were not found to be significantly different from one another using a Fisher’s z-test (z = −1.33, p = 0.18). It may be that the difference between the arrays is related to task difficulty. A paired t-test showed worse performance on the SAS array than the FHD array (SAS mean error = 10.35±4.23[SD]; FHD mean error = 8.83±3.50; t = −2.44, df = 23, p = 0.023).

The inter-rater comparison revealed a high level of relative agreement (n = 25, r = 0.925 p<0.001) and a slope close to 1 (line equation: y = 0.829x+1.398), indicating high absolute agreement.

## Discussion

We developed a novel method for measuring facial imitation that relies on the imitators’ capacity to make their expressions distinct from the other expressions in the set to be copied. In contrast to previous studies of imitation, our task sought to place demands on participants’ capacity for intentional control over their facial expression. Participants showed clear evidence of their ability to accurately imitate a range of emotional expressions, obtaining error scores that were significantly lower than chance level. Indeed, given the degree of similarity between adjacent stimuli, imitators performed remarkably well, and over 80% of ratings were correct within one step.

It might be asked whether participants achieved performance on the task through imitation or some other means. One objection may be that we cannot be sure that the expressions were novel to the participants, although novelty is a problematic criterion to use in defining imitation [38]. Also, could participants have used verbal labels to quantify the amount of emotion in each photograph? There are several reasons why this is unlikely. First, emotional attribution tends to be categorical [30] and the composite images of multiple emotions do not necessarily reflect any naturally occurring emotional state. Second, photographs were presented singly, each consisting of a blend of three emotions and shown alternately with the alternative blend of emotions. Therefore, to complete the task verbally would require participants to assign correct numerical values to the components involved and then to apply them in their pattern of expression. Close scrutiny of Figure 1 shows strong similarity of adjacent expressions and verbal description of these differences would be challenging. Even if it were the case that participants were using verbal labels to assist with imitation, this does not preclude it from being imitation. As discussed above, this experiment was designed drawing on the two-way method which has been established as the best experimental approach to testing for imitation, by determining whether participants show evidence of discriminating between two similar demonstrations of actions by showing a corresponding pattern of discrimination in their efforts to re-enact those actions. The task used here differed from mimicry by placing demands on the ability to control facial action intentionally, consistent with the definition of imitation of “performing an action by seeing how it is done” [8].

We hypothesised that an association would exist between empathy and imitation because both abilities would correlate with intentional control and motor planning capacity required for the expression of emotion, and since our accuracy measure relied on a capacity to form slightly different motor plans for slightly different emotional states, it provided a measure of this ability. It has been argued at least since Piaget [9] that imitation is distinguished from simpler sensory-motor integration by the use of representational mechanisms. Here, facial imitation required the formation of ‘secondary representations’ of actions [10], [39] in the form of motor plans that express emotional states. In simulation models of empathy [12], [17], [18], such models of emotional expression would be used for emotional understanding and may be formed by mapping codings for perceived actions onto motor planning systems involving mirror neuron mechanisms. Recent models of empathy have drawn a distinction between an approach relying on action-simulation using these mechanisms, and inferential approaches to mental state understanding [40]–[42]. Our findings would suggest that the EQ is sensitive to individual variability in the action-simulation aspects of empathic function. Nevertheless, other explanations for the association remain to be considered. It might also be suggested that the correlation with empathy stems from a greater ability to recognise emotion, rather than to imitate it. Research reports a weak relationship between empathy and emotion recognition. Groups of subjects known to have reduced empathy have also been shown to have reduced ability to recognise emotion, particularly in the ‘mind in the eyes task’ [43]. This includes sex-offenders [44] and those with autism [45], although, in this latter group the deficit may be subtle [46] or showing only a trend after controlling for IQ [47]. It has been suggested that this relationship could be mediated by alexithymia [48] which itself is also associated with low EQ scores [49]. We were able to identify only one recent study [50] that reported direct examination of the relationship between facial emotion recognition and EQ in a typical population. Fear was the only emotion where recognition correlated significantly with EQ scores, and then only with an eta-squared value of 0.11 (n = 135 participants), indicative of a small effect. In our study we found a stronger relationship with EQ and then with an emotion array that did not include fear. Of further interest is that the empathy relationship reported by Besel and Yuille only occurred at long (2 s) and not at brief (50 ms) exposures, which they suggest could be due to the role of more ‘cognitive’ as opposed to ‘automatic’ processes. Such ‘cognitive’ processes may perhaps be concerned with mental state representation that also occurs during imitation or intentional control over emotional expression. Therefore, it seems unlikely that recognition processes could solely account for the correlation in our experiment. Nevertheless, future work would benefit from an emotion recognition control task and attempts being made to distil the relative contributions of recognition, naming and re-enactment to the association between empathy and imitation. A final possibility to consider is that the association may have been mediated by a desire to please the experimenter. This could reflect variations in social motivation between individuals. This may also be a subject for examination in future studies.

It was interesting that we found little effect of visual feedback or practice in our study. Even combined, these two influences did not have a significant effect. The lack of these influences may relate to the likelihood that most people do not practise facial expressions in daily life, whether with or without mirrors (at least, not that we know of). Alternatively it may be that the study design was too brief to allow practice effects to emerge.

Our method had good inter-rater reliability, which is reassuring given that the rating stage could potentially provide a major source of variability. Whilst participants had many degrees of freedom in their generation of responses, raters’ choices were more limited through being required to fit the responses to the limited locations in the matrix. Raters also had plenty of time to decide where to allocate responses and quickly gained experience through repetition, enabling them to become sensitive to subtle differences between facial expressions.

In summary, the relationship between imitation and empathy could feasibly be mediated by one of, or a combination of several mechanisms that could all improve the mapping of a perceived action more accurately onto the motor plan for the same action. These may include action-perception mapping, secondary representation, heightened perception, verbal labelling, or social motivation. Further research will be required to explore these possibilities.

Despite the encouraging findings of this study, we would emphasise their preliminary nature. Most importantly, we report a novel experimental method developed to find a relatively simple and practical way of measuring facial imitation ability in an objective and reliable manner. Our method proved to be reliable and effective in distinguishing between participants according to their self-reported empathy which provided some evidence for our new method’s validity.
